# Serum and Urine Biomarkers for Human Renal Cell Carcinoma

**DOI:** 10.1155/2015/251403

**Published:** 2015-04-02

**Authors:** A. L. Pastore, G. Palleschi, L. Silvestri, D. Moschese, S. Ricci, V. Petrozza, A. Carbone, A. Di Carlo

**Affiliations:** ^1^Department of Medico-Surgical Sciences and Biotechnologies, Faculty of Pharmacy and Medicine, Urology Unit ICOT, Sapienza University of Rome, Via Franco Faggiana 1668, 04100 Latina, Italy; ^2^Department of Translational Medical Science, University of Naples “Federico II”, Via Sergio Pansini 5, 80131 Naples, Italy; ^3^Department of Medico-Surgical Sciences and Biotechnologies, Faculty of Pharmacy and Medicine, Pathology Unit ICOT, Sapienza University of Rome, Via Franco Faggiana 1668, 04100 Latina, Italy; ^4^Department of Medico-Surgical Sciences and Biotechnologies, Faculty of Pharmacy and Medicine, Sapienza University of Rome, Corso della Repubblica 79, 04100 Latina, Italy

## Abstract

Renal cell carcinoma (RCC) diagnosis is mostly achieved incidentally by imaging provided for unrelated clinical reasons. The surgical management of localized tumors has reported excellent results. The therapy of advanced RCC has evolved considerably over recent years with the widespread use of the so-called “targeted therapies.” The identification of molecular markers in body fluids (e.g., sera and urine), which can be used for screening, diagnosis, follow-up, and monitoring of drug-based therapy in RCC patients, is one of the most ambitious challenges in oncologic research. Although there are some promising reports about potential biomarkers in sera, there is limited available data regarding urine markers for RCC. The following review reports some of the most promising biomarkers identified in the biological fluids of RCC patients.

## 1. Background

Although advances in imaging techniques during the last decades have helped in the diagnosis of renal cell carcinomas, most of these malignancies are detected at an advanced stage due to their asymptomatic nature [1].

Renal cell carcinoma (RCC) is the ninth most common malignancy worldwide and even more common in developed countries (up to 6% of all cancers), with a peak of incidence occurring between 60 and 70 years of age, with a 3 : 2 male : female ratio [2, 3].

The urologic community is focusing on new therapeutic options, such as nephron-sparing surgical approaches and ablative procedures for smaller renal masses [4–7].

Since RCCs are often only incidentally diagnosed during imaging investigations for nonspecific symptoms or other abdominal disease, a more efficient early diagnosis technique is urgently required.

### 1.1. Risk Factors

In the risk evaluation of renal cell carcinoma, several genetic, clinical, and environmental factors have been identified [8–10]. Major risk factors include cigarette smoking (1/3 of all kidney cancers), alcohol use, hypertension, and obesity (with a linear correlation between increased body weight and RCC risk), as well as occupational and environmental factors (e.g., chronic exposure to cadmium, asbestos, petroleum products, ionizing radiations, and acetaminophen abuse) [11–13]. Patients with acquired cystic kidney disease (ACKD) appear to have an increased risk of developing RCC masses [14].

Renal carcinoma has also been described in association with numerous hereditary diseases (Von Hippel-Lindau disease, hereditary leiomyomatosis RCC, Birt-Hogg-Dubé syndrome, hereditary papillary RCC, hereditary nonpolyposis colorectal cancer syndrome, and autosomal dominant polycystic kidney disease) [15, 16].

### 1.2. Diagnosis

Diagnosis of renal cell carcinoma is often difficult since most renal masses remain asymptomatic until the late stages of the disease [1]. The classic triad of flank pain, palpable abdominal mass, and gross hematuria is rare (6–10%), and these signs typically arise at an advanced stage with aggressive histology [1].

Paraneoplastic syndromes are found in approximately 30% of patients with symptomatic RCCs. A few symptomatic patients present with symptoms caused by metastatic disease, such as bone pain or persistent cough [17].

When such clinical symptoms as well as arousing varicocele or bilateral lower extremity oedema are present, radiological investigation should be applied. Abdominal ultrasounds (US) are usually the first imaging procedure performed when RCC is suspected and enables investigators to distinguish between a benign kidney cyst and a tumor mass [18, 19].

CT imaging, before and after intravenous contrast, is useful for verifying the diagnosis and therefore obtaining information about the contralateral kidney (morphology and function). CT imaging also assesses tumor extension, including venous and lymph nodes involvement, as well as extrarenal spread [20]. In patients with possible venous involvement or proven allergy to intravenous contrast, MRI can be used [20]. Percutaneous renal tumour biopsies are increasingly being used for histological diagnosis of radiologic indeterminate renal masses [21].

### 1.3. Classification

The TNM classification system is generally recommended [22]. The latest version of the TNM classification was published in 2010 and this prognostic value of this classification has been confirmed by single and multi-institutional studies [23, 24]. However, some of the data remain controversial: T1 tumour subclassification with the current cut-off of 4 cm might not be adequately related to the widening of nephron-sparing surgery; some substages of the classification (pT2b, pT3a, pT3c, and pT4) may overlap [24]; the value of size stratification of T2 tumours has been (and continues to be) a debate [25]; tumours with renal sinus fat invasion are currently classified as pT3a; however, an increasing amount of data suggests that renal sinus fat invasion might be more closely related to a worse prognosis than perinephric fat invasion; therefore these different forms of fat invasions should not be classified in the same pT3a stage group [26].

In 2004, the World Health Organization issued a classification system for RCC, partially modified in 2013 by ISUP Vancouver Classification, which recognizes several histopathological entities. From a clinical point of view, three main types of RCC are the most important: clear cell (cRCC), papillary (pRCC), and chromophobe (chRCC) [27].

Clear cell carcinoma takes its name from its histologic features of a clear-cytoplasm cell surrounded by a dense endothelial network, and this type of cancer represents about 70% of all kidney cancers. Clear cell carcinoma is associated with loss of function of the VHL gene, which leads to hypoxia-inducible factors and Vascular Endothelial Growth Factor (VEGF), both strictly linked to tumor angiogenesis [28].

Papillary RCC (pRCC) is the second most common tumor and two different subtypes of pRCC have been identified and labelled as pRCC type 1 and pRCC type 2. pRCC type 1 is believed to be less aggressive than type 2. Diagnosis is mostly based on the papillary architecture. pRCC features a basophilic cytoplasm and foamy histiocytes are characteristic in this kind of neoplasm [29].

Chromophobe RCCs account for about 5% of RCCs and show the lowest risk of developing metastasis. Chromophobe RCCs often have an empty cytoplasm, perinuclear clearing, and a low mitotic rate [30]. Any subtype of RCC may feature a sarcomatoid dedifferentiation often associated with a more aggressive behaviour [31].

### 1.4. Treatment

Partial or radical nephrectomy remains the gold standard for the management of renal masses [32]. The use of an anatomical classification system for renal tumours has been introduced because it allows objective prediction of potential morbidity of nephron-sparing surgery and focal techniques. These systems include the assessment of anatomical features: tumour size, exophytic/endophytic properties, nearness to the collecting system and renal sinus, and anterior and posterior localisation. The widely used classifications include the Preoperative Aspects and Dimensions Used for an Anatomical (PADUA) score, the RENAL nephrometry score, and the C-index, which are currently used to standardize the description of renal tumours [31, 33, 34].

These classification systems are useful in planning patient's treatment and counselling and to specifically compare partial nephrectomy and tumour ablation case series. It should be noted that the anatomic scores, the clinical features, and the experience of the surgeon have to be considered together when making decisions about the best therapeutic strategy for each patient.

Since laparoscopic and robot-assisted radical nephrectomy has been proven to lead to lower morbidity than open surgical techniques, it has now become an established procedure [35]. A recently published prospective randomised clinical trial (RCT) compared radical with partial nephrectomy in solitary T1-2 N0M0 renal tumours <5 cm with normal contralateral kidney function. The cancer specific survival (CSS) was 98.5 after radical nephrectomy versus 97% after nephron-sparing surgery (NSS) [4].

Radical nephrectomy with complete removal of the interested kidney along with its perirenal fat and Gerota's fascia is currently recommended in patients not eligible for a nephron-sparing approach: a sparing procedure is not believed to be suitable for patients with an advanced tumor growth as well as a technically unfavourable localisation of the tumor itself [36]. When preoperative and intraoperative findings are normal, an adrenalectomy is not recommended [37].

Lymphadenectomy should only be considered for staging; in a recent prospective randomized study only 4% of cN0 patients had positive lymph nodes at final pathology, suggesting that LND represents overtreatment in the majority of cases [38].

Unfortunately, adjuvant therapies do not provide significant benefits in renal cell carcinoma patients who underwent surgery.

For patients unsuitable for surgical treatment due to advanced age or tumour stage, as well as comorbidity that leads to a higher surgical risk, minimally invasive techniques may be attempted. These procedures include ablative approaches such as radiofrequency ablation, including microwave ablation, cryoablation, and stereotactic radiation [6, 7].

Treatment for metastatic RCC consists of cytoreductive nephrectomy followed by systemic-drug therapy, which includes immunotherapeutic drugs, antiangiogenic agents, and mTOR inhibitors [39].

Nephrectomy of the primary tumor is curative only if all of the tumour deposits are removed. Thus, for patients with advanced metastatic RCC, nephrectomy is often palliative [40].

Chemotherapy is not considered effective as a systemic therapy after cytoreductive nephrectomy in metastatic RCC, while immunotherapy appears effective, although both interferon-alpha monotherapy and IL-2 monotherapy are believed to be inferior to targeted therapy. Despite this, IL-2 monotherapy continues to have a role in selected cases [41].

### 1.5. Prognostic Factors

Postoperative prognostic nomograms that combine different independent prognostic factors have been developed and validated [42, 43]. These systems seem to outperform TNM stage or Fuhrman grade alone for predicting survival. The most important advantage of these nomograms is related to their ability to measure predictive accuracy (PA), which evaluates all new predictive parameters. Every new prognostic system should demonstrate its PA superiority to conventional postoperative histoprognostic schemes, before being used [44]. Recently, new preoperative nomograms with excellent PAs have been designed and adopted [45].

## 2. Molecular Biomarkers

Histological analysis of TNM stage and the Fuhrman nuclear grade are the principal currently used independent prognostic factors for clinical work-up of patients. Although almost all tissue markers studies have been highly promising, almost all of these are based on retrospective series with small sample size and relatively short follow-up. The reliability of the assay used for marker detection represents another limitation of these studies. In fact, immunohistochemistry is semiquantitative and highly dependent on a range of variables, such as choice of antibody, antibody concentration, fixation techniques, variability in the interpretation and stratification criteria, and inconsistency in specimen handling and technical procedures. Therefore, the identification of molecular markers in body fluids (e.g., sera and urine), which can be used for screening, diagnosis, follow-up, and monitoring of drug-based therapy in RCC patients, is one of the most ambitious challenges in oncologic research [46, 47]. The global analysis of gene and protein expression profiles of biological specimens, like tissue, blood, or urine, is an emerging promising tool for new biomarker identification. The markers identified from this high throughput integrated “omics” technologies have promising potential but application in clinical diagnostics and practical improvement in disease management is extremely rare [48, 49]. Blood and urine are an ideal source of biomarker, for theoretical, methodological, and practical reasons [50]. Actually, there have been some promising reports about potential biomarkers in sera, but, to our knowledge, there is scant literature with regard to urine markers for RCC. As such, the available data are insufficient to justify routine clinical application. Here we summarize some of the most promising biomarkers for diverse clinical questions, identified in biological fluids of RCC patients (Tables 1 and 2).

### 2.1. Molecular Markers in Serum

There are currently no serum biomarkers available for accurate diagnosis of RCC. Some authors identified molecules that can be measured in serum by noninvasive tests. Using immunoassays, such as ELISA or immunonephelometry, or mass spectrometry, authors have detected altered production of serum proteins in RCC patients, which can provide useful diagnostic and/or prognostic information.

#### 2.1.1. Tumor Necrosis Factor Receptor-Associated Factor-1 (TRAF-1)

TRAF-1 is an adaptor protein involved in the regulation of cell survival, proliferation, differentiation, and stress responses. It has been reported that serum levels of TRAF-1 in RCC patients were significantly increased over control serum [51].

#### 2.1.2. Heat Shock Protein 27 (Hsp27)

Hypoxia, as for inflammation or oxidative stress, represents one of the main environmental stress conditions that can improve both tumor initiation and tumor progression. These conditions determinate, as a consequence, upregulation of some “stress proteins,” which take part in cellular stress response. Among these proteins, heat shock proteins play a relevant role as molecular chaperones for protein folding and unfolding. In particular, heat shock protein 27 (Hsp27) has been found to be hyperphosphorylated in many cancer types [52]. In a recent study, White et al. identified high levels of Hsp27 in both the serum and the urine of high-grade RCC patients [53].

#### 2.1.3. Inflammatory Proteins

Several studies have aimed to clarify the role of inflammatory immune cells and cytokines in tumorogenic processes of kidney cancer, in order to develop new immunotherapeutic strategies for the treatment of this disease. In liver cells, proinflammatory cytokines, such as IL-1, IL-6, and TNF-*α*, are able to control the synthesis of many other inflammation mediators, such as amyloid A, which is involved in the recruitment of immune cells to inflammatory sites and the induction of enzymes that degrade extracellular matrix [54]. Many authors, such as Mittal et al. and Fischer et al., identify serum amyloid A (SAA) as strong new potential biomarkers for RCC, which correlates positively to tumor stage, particularly in the advanced stages [55, 56].

Similar to SAA, levels of acute-phase C-reactive protein (CRP) increase within hours of inflammatory stimulus (e.g., microbial infection, trauma, infarction, autoimmune, or malignant diseases). In fact, de Martino et al. and Steffens et al. observed high serum CRP levels in RCC patients, associated with T classification and high-grade disease, with poor survival [57, 58].

Gamma-glutamyl transferase (GGT), an enzyme involved in glutathione metabolism and whose expression is often significantly increased in human malignancies, is another molecular marker of inflammation that is produced mostly in the liver but also in the kidneys, bile duct, pancreas, and spleen [59]. Hofbauer et al. identified GGT as a possible new independent prognostic factor for RCC patients management, as its levels increase in parallel to tumor staging and grading [60].

#### 2.1.4. Molecules Involved in Apoptosis

Tumorigenesis processes require alterations in physiological apoptotic mechanisms, with abnormal activity of antiapoptotic proteins or inhibition of proapoptotic mediators. In this way, cancer cells avoid apoptosis and continue to proliferate uncontrollably [61]. Among the main molecules involved in the programmed cell death, the tumor necrosis factor-related apoptosis-inducing ligand (TRAIL) has gained interest because it promotes extrinsic apoptosis preferentially in cancer compared to normal cells [62]. Toiyama et al. revealed a strong decrease of TRAIL serum levels in RCC patients, before surgery, compared to control, that was correlated to patients survival [63].

Moreover, in the circulation of cancer patients, it is possible to measure apoptosis-derived products, such as cytokeratins (CK), which are released into the blood in either intact or caspase-cleaved forms [64]. In particular, Yildiz et al. found increasing levels of serum M65 (the intact form of cytokeratin 18) in RCC patients compared to healthy participants, which could be predictive of their progression-free survival (PFS) [65].

#### 2.1.5. Molecules Related to Tumor Microenvironment

It has been wildly reported that tumor mass growth is strictly controlled by its microenvironment, in which components, such as fibroblasts and macrophages, release growth factors, cytokines, and angiogenic molecules to allow tumor progression. Hypoxia is an essential factor that characterizes the environment of tumor renal cells and tumor cells generally. Hypoxia promotes activation of hypoxia-inducible transcriptional factors (HIF-1*α* and HIF-1*β*) which improve expression of proangiogenetic factors, such as VEGF [66]. Moreover, genetic mutations of proteins involved in stabilization of HIF factors, such as Von Hippel-Lindau (VHL) protein, are considered the primary events responsible for the occurrence of the majority of clear cell RCC.

In addition to VHL protein, which is altered in 85% of RCC cases, the hypoxia-inducible factor prolyl hydroxylase-3 (PHD3) is frequently overexpressed in kidney cancer tissue [66]. PHD3 is one member of the PHD family involved in the degradation of hypoxia-inducible factor (HIF) proteins in cooperation with VHL. Tanaka et al. observed high levels of anti-PHD3 Ab titers in the serum of patients with RCC relative to healthy volunteers, and these levels decreased after surgical resection [67]. In addition, hypoxia is also able to promote expression of enzymes, such as carbonic anhydrases. These families of metalloenzymes are physiologically involved in acidification of gastric and renal tissues, but their synthesis has been reported to be altered in many tumor types [68]. In particular, Takacova et al. identified a higher level of carbonic anhydrase IX (CA IX) in both the tissue and the serum of cRCC patients, compared to non-cRCC patients. For this reason, this biomarker could represent a useful variable addictive to histological analysis [69].

#### 2.1.6. Miscellaneous

Nisman et al. detected significantly higher levels of two enzymes involved in tumor cells metabolism, pyruvate kinase isoenzyme type M2 (TuM2-PK), and thymidine kinase 1 (TK1), in RCC patients sera, compared to controls, and this increase is positively associated with tumor stage and/or grade [70].

In RCC, as in other cancer types, alteration of physiological ubiquitin proteasome system (UPS) mechanisms has been observed [71]. de Martino et al. observed an increase in serum levels of 20S proteasome in RCC patients, compared to healthy subjects, which are strictly related to their disease-specific survival (DSS) [72].

Furthermore, about 10% of RCCs feature calcifications in peritumoral regions. For this reason the measurement of blood calcium levels is a key analysis used for clinical diagnosis of RRC. In many cases, alterations of hypercalcemia in RCC are due to changes in the metabolism of inhibitors and promoters of calcification, such as Fetuin A, Osteopontin (OPN), and Osteoprotegerin [73]. Papworth et al. showed that serum OPN was significantly higher in patients with papillary RCC compared to other RCC histotypes and that OPN levels were positively correlated with TNM stage and nuclear grade [74].

### 2.2. Molecular Markers in Urine

The idea of following localized tumors or monitoring drug-based therapy results by simply analyzing tumor-specific markers in the easily available excretory products of the kidney is desirable. However, to the best of our knowledge, there is only scant literature addressing urine markers for RCC.

#### 2.2.1. Nuclear Matrix Proteins-22 (NMP-22)

Nuclear matrix proteins are part of the internal framework of the nucleus and are known to have important roles in DNA replication, transcription of RNA, and regulation of gene expression. The characteristics of nuclear matrix proteins make them a potential marker of malignant cells associated with the abnormal distribution of genetic material and increased mitosis. Several of the NMPs are organ specific. Cancer-specific NMPs have been identified in breast, colon, bone, and urothelium, and NMP-22 has been recognized as a potential urothelial-specific cancer marker. The prostate-specific antigen (PSA) for prostate cancer and the urinary nuclear matrix protein-22 (NMP-22) are the only Federal Drug Administration- (FDA-) approved screening markers. Urinary NMP-22 is known to be specific for transitional cell carcinoma [75], and a rapid flow-through diagnostic test is available. There are some reports suggesting that NMP-22 might work as an RCC screening marker [76–78]. Kaya et al. found that in RCC patients, urinary NMP-22 levels were significantly higher than those observed in patients with kidney stones and simple renal cysts used as control group [76]. Similarly, Ozer et al. and Huang et al. reported that preoperatively urinary NMP-22 levels were significantly higher in each of their respective RCC groups than in subjects with benign renal conditions [77, 78].

#### 2.2.2. Neutrophil Gelatinase-Associated Lipocalin (NGAL)

Lipocalin 2, also known as Neutrophil Gelatinase-Associated Lipocalin (NGAL), is a prominent member of the lipocalin family. The lipocalins constitute a large group of small predominantly extracellular proteins previously regarded as obscure transporters of hydrophobic ligands ([79, 80] and ref. therein). In humans, NGAL was originally identified as a 25 kDa protein covalently linked to matrix metalloproteinase-9 (MMP-9) in neutrophils, which normally provide the main cellular source of circulating NGAL. By forming the MMP-9/NGAL complex, NGAL protects MMP-9 from proteolytic degradation, increasing the enzymatic activity of MMP-9 and subsequently enhancing tumoral invasiveness and diffusion [81]. NGAL is expressed in the epithelial cells of the kidney and is involved in kidney development. NGAL is a biomarker of tubular injury and is expressed in several histotypes of renal tumor, and its high expression is associated with higher histological grade of cRCC, and papillary RCC, whereas oncocytoma and urothelial carcinoma exhibit lower expression levels. It is evident that any NGAL systematically released from malignant transformed cells would be freely filtered by the kidney glomerulus; however, these may be expected to be largely reabsorbed by efficient endocytic mechanisms in proximal tubules. Therefore, urinary excretion of NGAL is more likely to be present in tandem with a concomitant renal tubular injury that increases* de novo* NGAL synthesis and/or precludes NGAL reabsorption. Di Carlo and Morrissey et al. showed that in cRCC patients the mean value of urinary NGAL was higher than that observed in the urine of control group. However, no correlation with tumor size or stage was identified in either study [82, 83].

#### 2.2.3. Kidney Injury Molecule-1 (KIM-1)

Kidney injury molecule-1 (KIM-1) is a urinary biomarker of diagnostic relevance in a wide variety of acute and chronic kidney diseases. However, although KIM-1 has diagnostic sensitivity for kidney cancer, it is well known to reflect many types of kidney injuries, thus limiting its specificity as a diagnostic marker for renal cancer [83].

#### 2.2.4. Matrix Metalloproteinases (MMPs)

The matrix metalloproteinases (MMPs) are a large group of zinc-containing endopeptidase with a central role in the degradation of all types of extracellular matrix (ECM) components. They are involved in direct and indirect release of growth factors enhancing tumor growth and tumorigenicity. In particular, MMP-2 and MMP-9 (also known as gelatinase-A and gelatinase-B) are most linked to the malignant phenotype of tumor cells and are commonly used as markers of malignant cancer. However, despite the tissue evidence, serum and urine MMP activity seems to be inadequate for identifying renal cancer ([84] and ref. therein).

#### 2.2.5. Aquaporin-1 (AQP-1) and Perilipin 2 (PLIN2)

It has been shown that aquaporin-1 (AQP-1) and adipocyte-differentiation-related protein (renamed Perilipin 2 (PLIN2)) are overexpressed in surgical excised renal tumor tissue. On the basis of the potential for urinary excretion or elimination of these upregulated proteins, Morrissey et al. found an elevated urine concentration of AQP-1 and PLIN2 in patients with clear cell and papillary carcinoma, as compared with controls [85]. Moreover, the urinary concentrations of these two markers were not significantly influenced by noncancerous kidney diseases, such as diabetic nephropathy, urinary tract infection, and glomerulonephritis [86], and reflected the tumor size and stage [87].

## 3. Conclusions

Currently, there is no diagnostic modality for early detection of renal cancer, other than incidental radiologic discovery, and no method of surveillance of recurrence or response to chemotherapy. Biomarkers are easily measured substances that can be used to monitor normal and abnormal biologic function. Unfortunately, there is no existing biomarker for kidney cancer diagnosis. The currently available biomarkers appear to have the most utility as diagnostic adjuncts, as prognostic indicators, and in following up patients.

## Figures and Tables

**Table 1:** Potential serum biomarkers in RCC.

Marker	Author (yr)	RCC (number)	HC (number)	RCC versus HC	Stage	Grade
TRAF-1	Rajandram et al. (2014) [51]	15	15	↑		
Hsp27	White et al. (2014) [53]	54	36	↑		
SAA	Mittal et al. (2012) [55]	422			↑	
	Fischer et al. (2012) [56]	115	24	↑		
hsCRP	de Martino et al. (2013) [57]	41			↑	↑
	Steffens et al. (2012) [58]	1.161			↑	
GGT	Hofbauer et al. (2014) [60]	921			↑	↑
TRAIL	Toiyama et al. (2013) [63]	84	52	↓		
M-65	Yildiz et al. (2013) [65]	39	39	↑		
Ab anti-PHD3	Tanaka et al. (2011) [67]	22	26	↑		
CA IX	Takacova et al. (2013) [69]	74			↑	
TuM2-PK	Nisman et al. (2010) [70]	116	20	↑	↑	↑
TK1	Nisman et al. (2010) [70]	116	20	↑	↑	
20S proteasome	de Martino et al. (2012) [72]	113	15	↑		
OPN	Papworth et al. (2013) [74]	269			↑	

RCC: renal cell carcinoma; HC: healthy control; No: number; TRAF-1: tumor necrosis factor receptor-associated Factor-1; Hsp27: heat shock protein *β*1; GGT: Gamma-glutamyl transferase; M65: intact form of CK18; TRAIL: tumor necrosis factor-related apoptosis inducing ligand; CA IX: carbonic anhydrase IX; hsCRP: high sensitivity C-reactive protein; SAA: serum amyloid A; OPN: osteopontin; anti-PHD3 Ab: anti-hypoxia-inducible factor prolyl hydroxylase-3 antibody; TuM2-PK: pyruvate kinase type M2; TK1: thymidine kinase 1.

**Table 2:** Potential urinary biomarkers in RCC.

Marker	Author (yr)	RCC versus CG
NMP-22	Kaya et al. (2005) [76]	↑
	Ozer et al. (2002) [77]	↑
	Huang et al. (2000) [78]	↑
NGAL	Di Carlo (2013) [82]	↑
	Morrissey et al. (2011) [83]	↑
KIM-1	Morrissey et al. (2011) [83]	↑
MMPs	di Carlo (2012) [84]	↑
AQP-1	Morrissey and Kharasch (2013) [86]	↑
AQP-1	Morrissey et al. (2014) [87]	↑
PLIN2	Morrissey and Kharasch (2013) [86]	↑
PLIN2	Morrissey et al. (2014) [87]	↑

RCC: renal cell carcinoma; CG: control group; NMP-22: nuclear matrix protein-22; KIM-1: kidney injury molecule-1; MMP: matrix metalloproteinases; AQP-1: aquaporin-1; PLIN2: Perilipin 2.
